# Exploring a four-gene risk model based on doxorubicin resistance-associated lncRNAs in hepatocellular carcinoma

**DOI:** 10.3389/fphar.2022.1015842

**Published:** 2022-11-10

**Authors:** Zunyi Zhang, Weixun Chen, Chu Luo, Wei Zhang

**Affiliations:** Hepatic Surgery Center, Tongji Hospital, Tongji Medical College, Huazhong University of Science and Technology, Wuhan, China

**Keywords:** hepatocellular carcinoma, long non-coding RNAs, RNF157-AS1, risk model, lncRNAs

## Abstract

**Background:** Liver cancer is a lethal cancer type among which hepatocellular carcinoma (HCC) is the most common manifestation globally. Drug resistance is a central problem impeding the efficiency of HCC treatment. Long non-coding RNAs reportedly result in drug resistance. This study aimed to identify key lncRNAs associated with doxorubicin resistance and HCC prognosis.

**Materials and Methods:** HCC samples with gene expression profiles and clinical data were accessed from public databases. We applied differential analysis to identify key lncRNAs that differed between HCC and normal samples and between drug-fast and control samples. We also used univariate Cox regression analysis to screen lncRNAs or genes associated with HCC prognosis. The least absolute shrinkage and selection operator (LASSO) was used to identify the key prognostic genes. Finally, we used receiver operating characteristic analysis to validate the effectiveness of the risk model.

**Results:** The results of this study revealed RNF157-AS1 as a key lncRNA associated with both doxorubicin resistance and HCC prognosis. Metabolic pathways such as fatty acid metabolism and oxidative phosphorylation were enriched in RNF157-AS1-related genes. LASSO identified four protein-coding genes—*CENPP*, *TSGA10*, *MRPL53*, and *BFSP1*—to construct a risk model. The four-gene risk model effectively classified HCC samples into two risk groups with different overall survival. Finally, we established a nomogram, which showed superior performance in predicting the long-term prognosis of HCC.

**Conclusion:** RNF157-AS1 may be involved in doxorubicin resistance and may serve as a potential therapeutic target. The four-gene risk model showed potential for the prediction of HCC prognosis.

## Introduction

Primary liver cancer is the seventh most frequently diagnosed cancer worldwide and shows a high mortality rate among all cancer types (Sung et al., 2021). Asia and Africa are two regions with large numbers of cases (Sung et al., 2021). Hepatocellular carcinoma (HCC) accounts for 85–90% of all cases of liver cancer (McGlynn et al., 2021). Hepatitis B virus (HBV) infection largely contributes to the development of HCC; other factors such as hepatitis C virus (HCV) infection, alcohol use, nonalcoholic fatty hepatitis, and liver cirrhosis can also increase the risk of HCC. Surgical treatment is a recognized strategy for HCC treatment, in which hepatectomy and liver transplantation are two major methods to increase survival time (Zhou et al., 20192020). However, for patients with metastatic HCC, surgical treatment is not effective. Chemotherapy or radiotherapy is the main treatment choice for killing metastatic cancer cells.

Drugs for systemic chemotherapy, including sorafenib, lenvatinib, and oxaliplatin, have demonstrated effectiveness as first-line therapy against late-stage HCC in clinical studies and are included in treatment guidelines (Zhou et al., 20192020). However, the use of single drugs is unsatisfactory and drug resistance is common with the continuous use of these drugs (Lohitesh et al., 2018). For example, only around 30% of patients with HCC can benefit from sorafenib and show only 6 months of drug response (Tang et al., 2020). Therefore, understanding drug resistance is required to optimize these chemotherapeutic drugs. Recent studies suggest the roles of epigenetics, regulated RNAs, cell death, and the tumor microenvironment in the occurrence of drug resistance in HCC (Ding et al., 2018; Liang et al., 2020; Tang et al., 2020).

Long non-coding RNAs (lncRNAs) are involved in multiple biological or pathological processes regulating cell proliferation, cell death, immune response, and others, which also contribute to drug resistance in HCC (Ding et al., 2018). For example, the knockdown of lncARSR inhibited PTEN expression and activated the PI3K/Akt pathway during the treatment of HCC, which was involved in doxorubicin resistance (Li et al., 2017). Tsang and Kwok reported that H19 knockdown suppressed MDR1 expression and increased HepG2 cell sensitivity to doxorubicin (Tsang and Kwok, 2007). Zhou et al. (2017)observed that the knockdown of HOTAIR lncRNA increased cisplatin efficiency on HCC cells by regulating the STAT3/ABCB1 signaling pathway. Therefore, lncRNAs play a critical role in drug resistance and HCC progression, and it is crucial to identify lncRNAs related to doxorubicin resistance to guide HCC treatment. Therefore, in the current study, we aimed to identify key lncRNAs related to drug resistance in HCC based on expression profiles of HCC and to construct a lncRNA-related risk model to predict HCC prognosis.

## Materials and methods

### Datasets

The Cancer Genome Atlas-Liver Hepatocellular Carcinoma (TCGA-LIHC) dataset, which contains RNA sequencing (RNA-seq) data and clinical information from HCC and paracancerous samples, was downloaded through the TCGA GDC API. The GSE76427 and GSE125180 datasets were downloaded from Gene Expression Omnibus (GEO) (https://www.ncbi.nlm.nih.gov/geo/query/acc.cgi?acc=GSE76427, https://www.ncbi.nlm.nih.gov/geo/query/acc.cgi?acc=GSE125180). The GSE76427 dataset contains microarray data from HCC samples, while the GSE125180 dataset contains microarray data from three doxorubicin drug-fast samples and three control samples.

### RNA-seq and microarray data processing

For RNA-seq data in the TCGA dataset, we included samples with data on overall survival and survival status. The Ensembl ID of each gene was transferred to the gene symbol. We determined the average expression level for genes with multiple gene symbols. After processing, 360 primary HCC samples and 50 paracancerous samples remained.

For microarray data in the GEO datasets, normal samples were removed. Probes were converted to gene symbols using the platform annotation file. Probes corresponding to multiple genes were removed. Samples lacking data on survival time and survival status in the GSE76427 dataset were excluded. Finally, 115 HCC samples from the GSE76427 dataset remained.

### Acquisition of lncRNA data

Gene transfer format (GTF) file (v32) was obtained from the GENCODE website (https://www.gencodegenes.org/). The expression data from the TCGA and GSE76427 datasets were converted to mRNA and lncRNA data. Mutual lncRNA data from the two datasets were included in the analyses.

### Identification of key lncRNAs related to drug resistance and HCC prognosis

The limma R package (Ritchie et al., 2015) was applied to perform differential analysis and identify differentially expressed lncRNAs (DElncRNAs) between HCC and paracancerous samples in the TCGA dataset, drug-fast and control samples of GSE76427. *p* < 0.05 and |log2(fold change)|>log2(1.2) (Chen et al., 2021) were determined to screen DElncRNAs. The intersection of DElncRNAs in two datasets was included, and univariate Cox regression analysis was further performed on the screened DElncRNAs, with *p* < 0.05 indicating potential prognostic DElncRNAs.

### Pathway analysis

We downloaded the Kyoto Encyclopedia of Genes and Genomes (KEGG) pathways from the KEGG website (https://www.genome.jp/kegg/pathway.html). Single-sample gene set enrichment analysis (ssGSEA) was performed for each HCC sample in the TCGA dataset using the ssGSEA algorithm in the GSVA R package (Hänzelmann et al., 2013).

### Evaluation of tumor microenvironment and the relationships between DElncRNAs and immune infiltration

We used CIBERSORT (Chen et al., 2018) to estimate the proportions of 22 immune-related cells and the ESTIMATE tool (Yoshihara et al., 2013) to calculate the immune and stromal scores. We applied the Hmisc R package (https://cran.r-project.org/web/packages/Hmisc/index.html) to conduct Pearson correlation analyses to assess the correlations between DElncRNA expression and immune infiltration. WebGestaltR package (Liao et al., 2019) was used to perform enrichment analysis on gene ontology (GO) and KEGG pathways using a false discovery rate (FDR) of <0.05 to screen for enriched GO terms and pathways.

### Construction of a risk model related to key DElncRNAs

We first performed Pearson correlation analysis using the “rcorr” function in the Hmisc R package to screen for genes significantly correlated with the key DElncRNAs (|correlation coefficient| > 0.4 and *p* < 0.001). Next, we used the survival R package to perform a univariate Cox regression analysis of the screened genes, with genes with *p* < 0.001 defined as potential prognostic genes. Least absolute shrinkage and selection operator (LASSO) Cox regression analysis was conducted using the glmnet R package (Friedman et al., 2010) to decrease the number of prognostic genes. For each variable (prognostic gene), a trajectory of coefficient variation with the changing lambda value was visualized. With increasing lambda values, the coefficients of prognostic genes approached zero. We applied ten-fold cross-validation to construct the model, which was defined as follows: Risk Score = Σβi×Expi, where βi represents the Cox coefficients of the prognostic genes and Expi represents the expression levels. For each sample, a risk score was calculated and converted to a z-score. According to z-score = 0, the samples were divided into high-risk (z-score > 0) and low-risk (z-score < 0) groups. We conducted Kaplan–Meier survival analysis to draw survival plots of the two risk groups. The TimeROC R package (Blanche et al., 2013) was used to examine the efficiency of the risk model in predicting overall survival.

### Statistical analysis

The bioinformatics analysis in this study was supported by the Sangerbox tool (http://vip.sangerbox.com/) (Shen et al., 2022). All statistical analyses were conducted in R software (v4.1). Wilcoxon tests were performed for comparisons between groups. Log-rank tests were used in the Cox regression and survival analyses. *p* < 0.05 was defined as statistical significance.

## Results

### Screening lncRNAs related to both drug sensitivity and HCC prognosis

**FIGURE 1:**
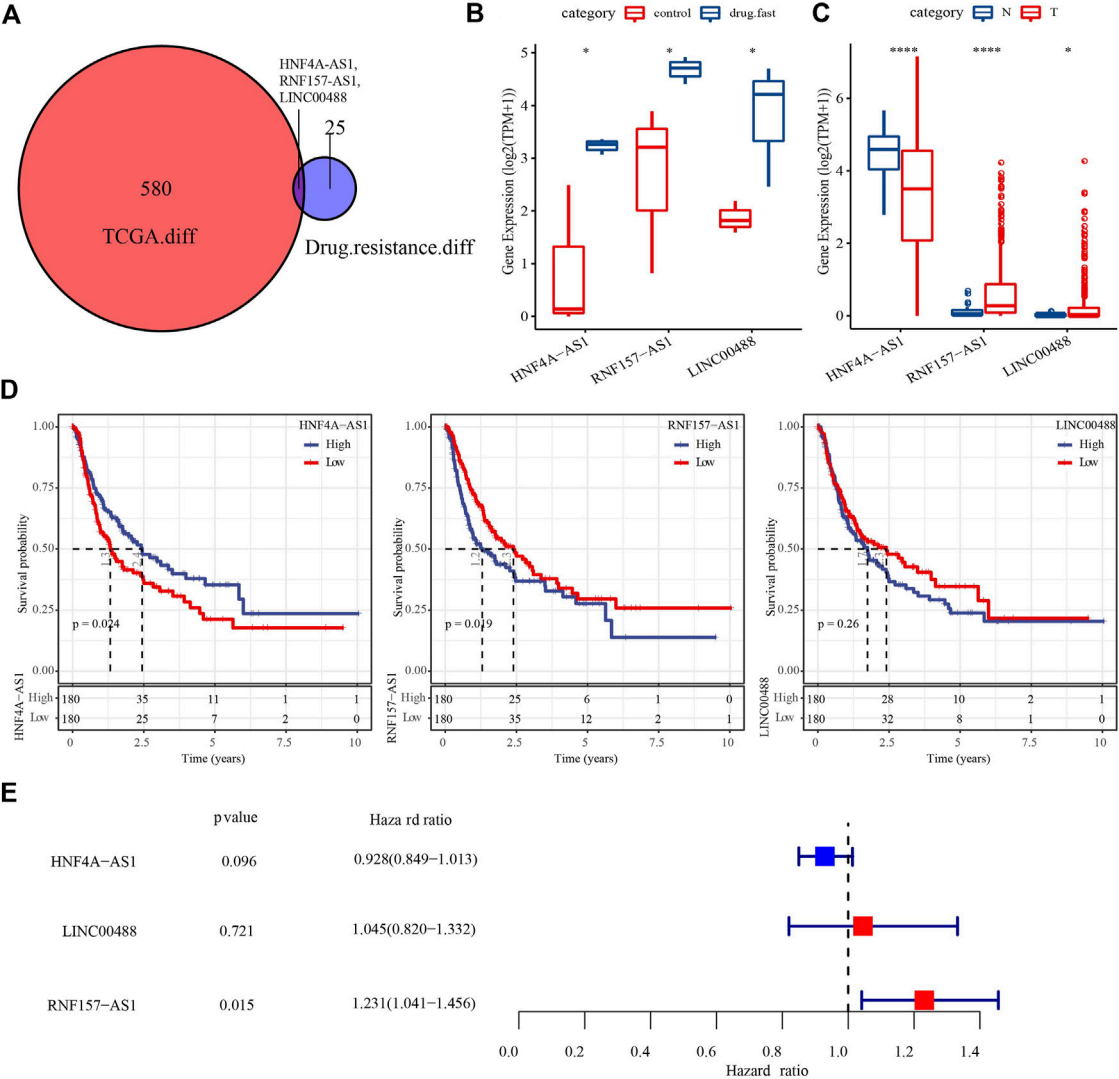
Identification of key DElncRNAs related to drug resistance and HCC prognosis. **(A)** Venn plot of DElncRNAs in the TCGA and GSE125180 datasets. **(B)** Expression of three DElncRNAs in control and drug-fast samples from the GSE125180 dataset. Wilcoxon tests were conducted. **(C)** Expression of three DElncRNAs in HCC and paracancerous samples in the TCGA dataset. Wilcoxon tests were conducted. **(D)** Kaplan–Meier survival plots of high- and low-expression of three DElncRNAs. **(E)** Univariate Cox regression analysis of three DElncRNAs. Log-rank tests were conducted. *p < 0.05, ****p < 0.0001.

We first screened for DElncRNAs between the drug-fast and control groups in the GSE125180 dataset by differential analysis. We identified a total of 28 DElncRNAs, including 18 that were upregulated and 10 that were downregulated (*p* < 0.01, |log2(fold change)| > log2(1.2); Supplementary Figure S1A). We then performed the same analysis between HCC and paracancerous samples in the TCGA dataset, which revealed 534 upregulated and 49 downregulated DElncRNAs (Supplementary Figure S1B). The Venn plot showed that three DElncRNAs (HNF4A-AS1, RNF157-AS1, and LINC00488) were found in both datasets (Figure 1A). The expression of the three lncRNAs was upregulated in the drug-fast group compared to that in the control group (*p* < 0.05; Figure 1B). These lncRNAs also displayed differential expression levels between HCC and paracancerous samples (*p* < 0.05; Figure 1C). To explore the relationship between three lncRNAs and HCC prognosis, we classified the HCC samples as having high or low expression based on the median cut-off value of the expression of the three lncRNAs. Survival analysis revealed that the high- and low-expression groups of HNF4A-AS1 and RNF157-AS1 had differential overall survival (*p* < 0.05; Figure 1D). However, univariate Cox regression analysis showed that only RNF157-AS1 expression was independently associated with overall survival (*p* = 0.015, hazard ratio (HR) = 1.231; Figure 1E).

### Potential pathways related to RNF157-AS1

**FIGURE 2:**
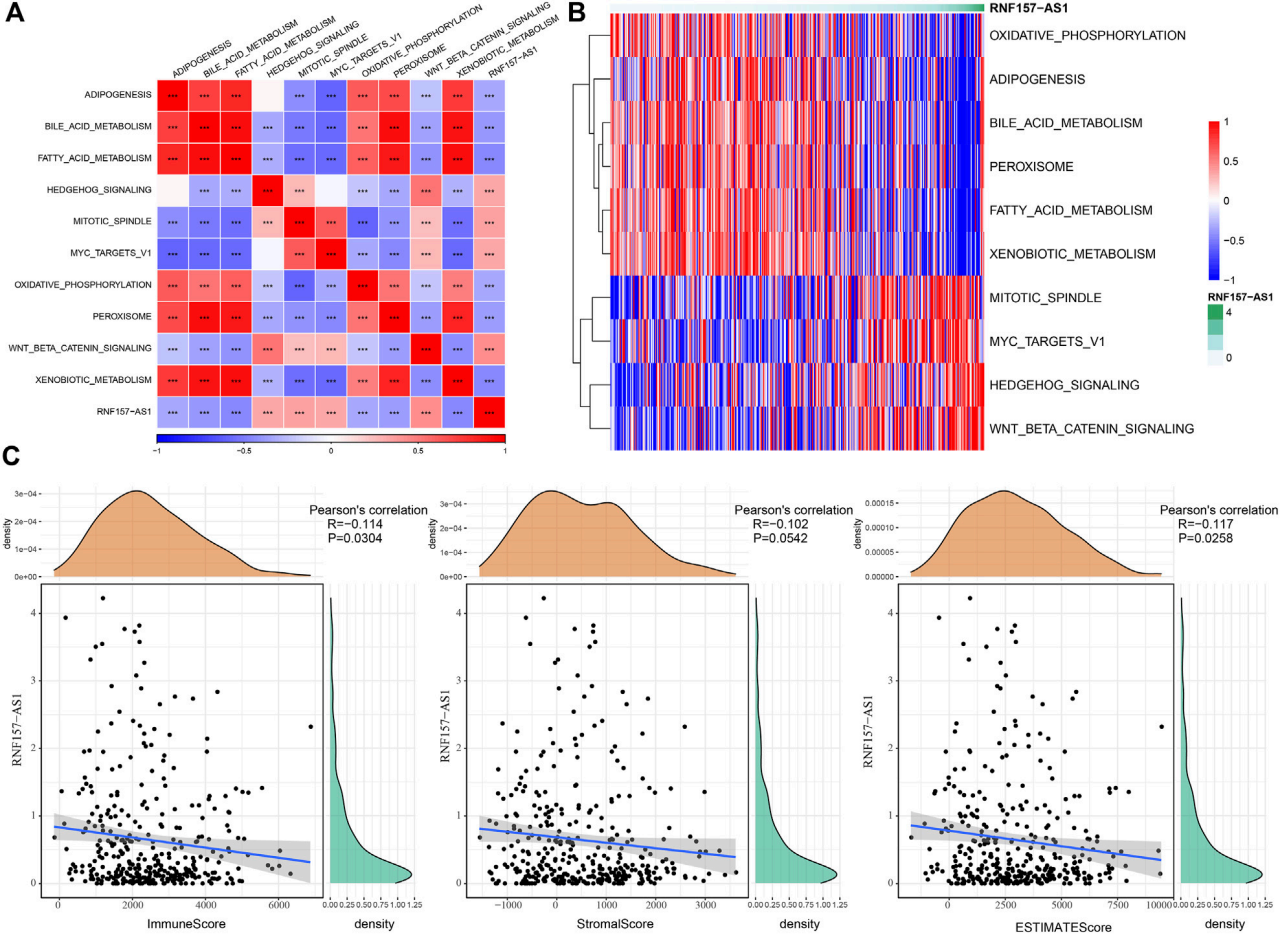
Functional pathways related to RNF157-AS1 and the relationship between RNF157-AS1 and immune infiltration. **(A)** Correlation analysis between the enrichment of pathways and RNF157-AS1 expression. Red and blue indicate positive and negative correlations, respectively. **(B)** Heatmap showing the enrichment score of pathways ranked by increasing RNF157-AS1 expression. Darker green: higher expression. Red and blue: high and low expression levels, respectively. **(C)** Pearson correlation analysis of RNF157-AS1 with immune, stromal, and ESTIMATE scores.

As we identified that RNF157-AS1 expression was dysregulated in drug-fast and HCC samples and was also an independent risk factor, we considered RNF157-AS1 as an important lncRNA involved in HCC development and drug resistance. Therefore, we further explored the functional pathways with which it was correlated. The enrichment score for all hallmark pathways was calculated for each HCC sample. We further performed a correlation analysis between the pathway score and the RNF157-AS1 expression level. We identified 10 functional pathways that were associated with RNF157-AS1 expression (Figure 2A). For example, bile acid metabolism, fatty acid metabolism, and oxidative phosphorylation were negatively associated with RNF157-AS1 expression, while MYC target V1 and WNT-β catenin signaling were positively associated with RNF157-AS1 expression. A heatmap also showed the correlations between the 10 pathways and RNF157-AS1 expression, consistent with Figure 2A (Figure 2B).

### Association between RNF157-AS1 and immune infiltration

The characteristics of the tumor microenvironment reflect cancer development and prognosis, as well as the sensitivity to cancer treatment. Therefore, we assessed the potential correlation between RNF157-AS1 and the tumor microenvironment in HCC. The CIBERSORT results indicated that M2 macrophages, CD8 T cells, and gamma delta T cells were negatively correlated with RNF157-AS1 expression, while naïve CD4 T cells, M0 macrophages, eosinophils, and neutrophils were positively correlated with RNF157-AS1 expression (*p* < 0.05; Supplementary Figure S2A). The results of the ESTIMATE analysis further demonstrated a significant correlation between RNF157-AS1 expression and immune infiltration (Figure 2C). In addition, we calculated the ssGSEA scores of immune-related pathways and discovered that complement and coagulation cascades, natural killer cell-mediated cytotoxicity, and FC gamma-R-mediated phagocytosis were associated with RNF157-AS1 expression (Supplementary Figure S2B). Together, these results suggested that RNF157-AS1 was involved in immune infiltration regulation through some immune-related pathways.

### Identification of prognostic genes related to RNF157-AS1

**FIGURE 3:**
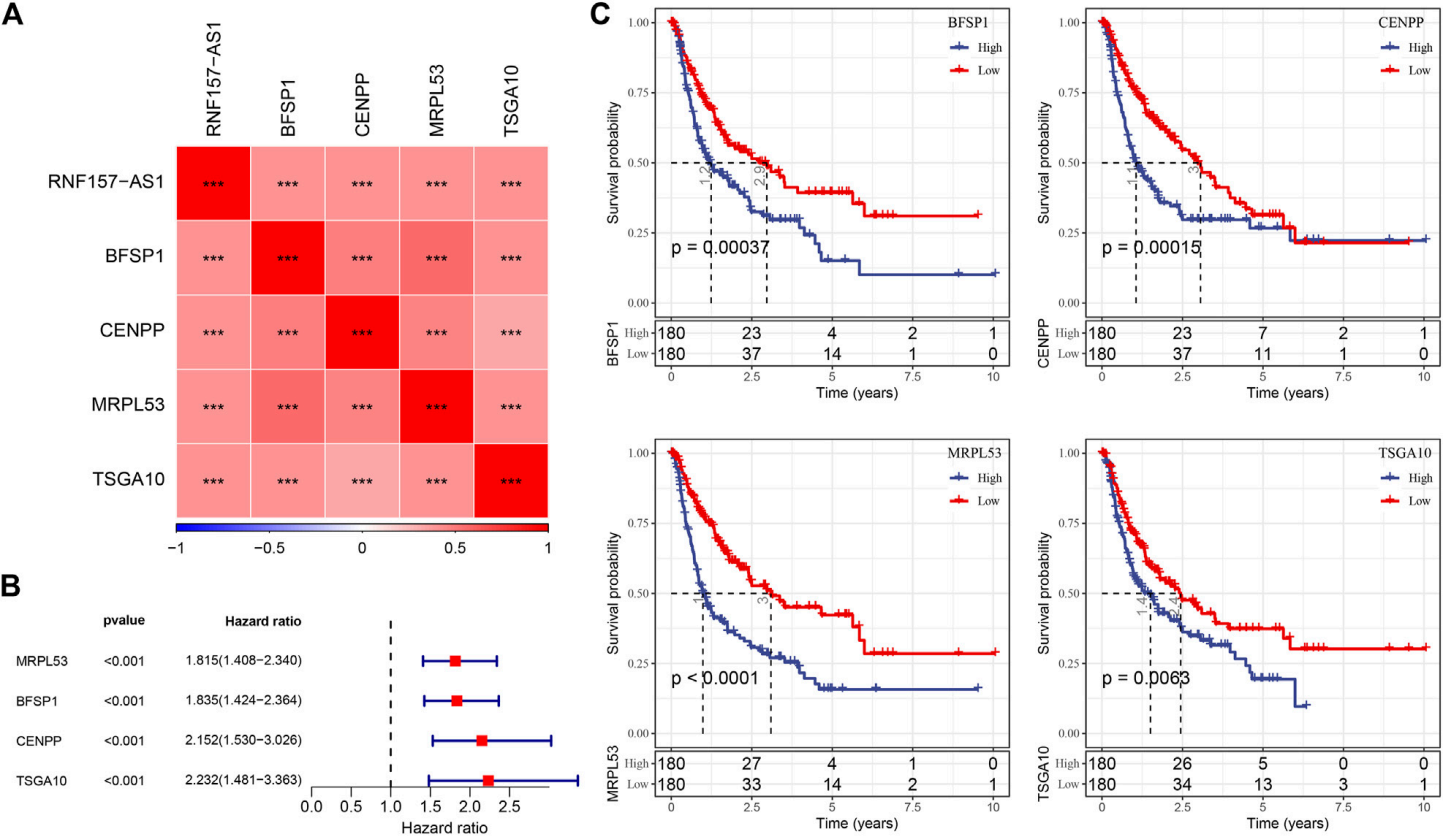
Relationship of RNF157-AS1 with four key PCGs and the association between four PCGs and HCC prognosis. **(A)** Pearson correlation analysis between the expression of RNF157-AS1 and four lncRNAs. Red and blue: positive and negative correlations, respectively. **(B)** Univariate Cox regression analysis of four lncRNAs. **(C)** Kaplan–Meier survival plots of high- and low-expression groups in the four lncRNAs. Log-rank tests were conducted. ***p < 0.001.

We performed Pearson correlation analysis to identify protein-coding genes (PCGs) associated with RNF157-AS1 based on their expression levels. The results revealed 1,498 PCGs that were significantly associated with RNF157-AS1 (correlation coefficient |>0.4, *p* < 0.001). Functional analysis revealed enrichment of mRNA-related terms, cell cycle, and RNA transport in the 1,498 PCGs (Supplementary Figures S3A–D). Univariate Cox regression analysis to identify prognostic genes identified 166 risk genes that were associated with HCC prognosis (Supplementary Figure S4A). Subsequently, we implemented LASSO regression analysis to determine the key prognostic genes for constructing an optimal risk model. At a lambda value of 0.0708, the model reached the optimal status (Supplementary Figures S4B,C). Finally, four risk genes remained, including *CENPP*, *TSGA10*, *MRPL53*, and *BFSP1* (Supplementary Figure S4D). The expression levels of these four genes were related to RNF157-AS1 expression (*p* < 0.001; Figure 3A). Cox regression analysis showed that the four genes were independent risk factors (*p* < 0.001, HR > 1.8; Figure 3B). Survival analysis showed distinct overall survival between the high- and low-expression groups of all four genes (*p* < 0.01; Figure 3C), indicating that these four genes were highly associated with HCC prognosis. Therefore, we included these genes in the construction of the risk model.

### Validation of the four-gene risk model

**FIGURE 4:**
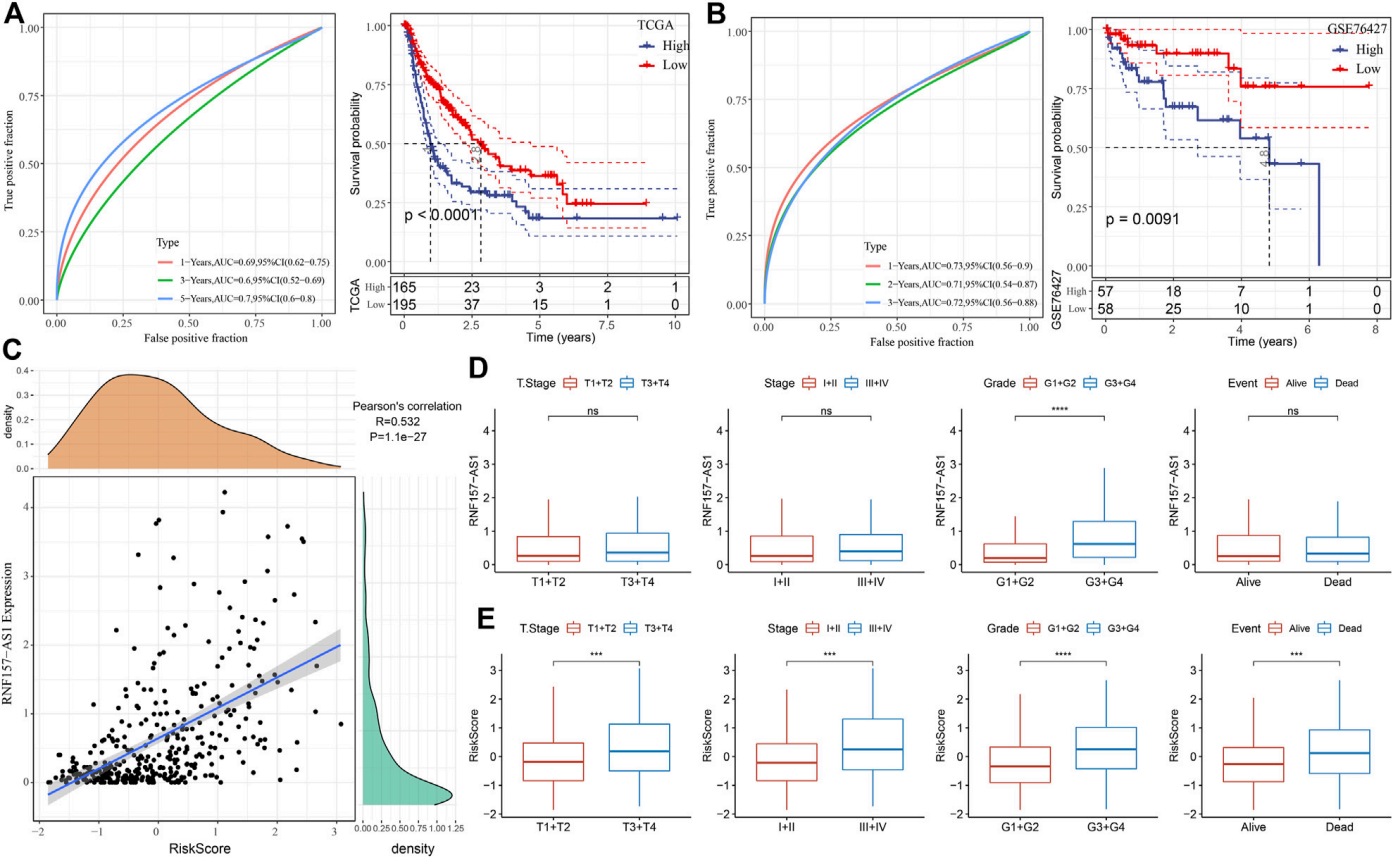
Performance of the four-gene risk model. **(A)** ROC analysis of survival plots of the risk model in the TCGA dataset. **(B)** ROC analysis of survival plots of the risk model in the GSE76427 dataset. **(C)** Pearson correlation analysis between RNF157-AS1 expression and risk score. **(D)** RNF157-AS1 expression of HCC samples grouped by different clinical features. **(E)** Distribution of risk scores for different clinical features. Log-rank tests were conducted in **(A, B)**. Wilcoxon tests were performed in **(D, E)**. ns: no significance. ***p < 0.01, ****p < 0.0001.

For each HCC sample, we calculated the risk score and validated the efficiency of the risk model in predicting prognosis by ROC analysis. The results showed that the risk model had a good performance in evaluating 1- and 5-year prognosis, with AUC values of 0.69 and 0.70, respectively, in the TCGA dataset (Figure 4A). HCC samples were classified into two risk groups (high and low risk) based on the cut-off (z-score = 0). The Kaplan–Meier survival plots showed a significant difference in the prognosis of the two risk groups (*p* < 0.0001; Figure 4A). In the GSE76427 dataset, we observed similar results, which suggested the robust performance of the risk model (Figure 4B). Notably, we observed a markedly positive correlation between RNF157-AS1 expression and risk score (R = 0.532; Figure 4C). We then evaluated the distribution of RNF157-AS1 expression levels and risk scores in different clinical features. The RNF157-AS1 expression level was much higher in G3 and G4 compared to that of G1 and G2 (*p* < 0.0001; Figure 4D). Moreover, severe clinical stages had higher risk scores than moderate stages (*p* < 0.001; Figure 4E), suggesting the reliability of the risk model.

### Establishing a nomogram based on the four-gene risk model and clinical features

**FIGURE 5:**
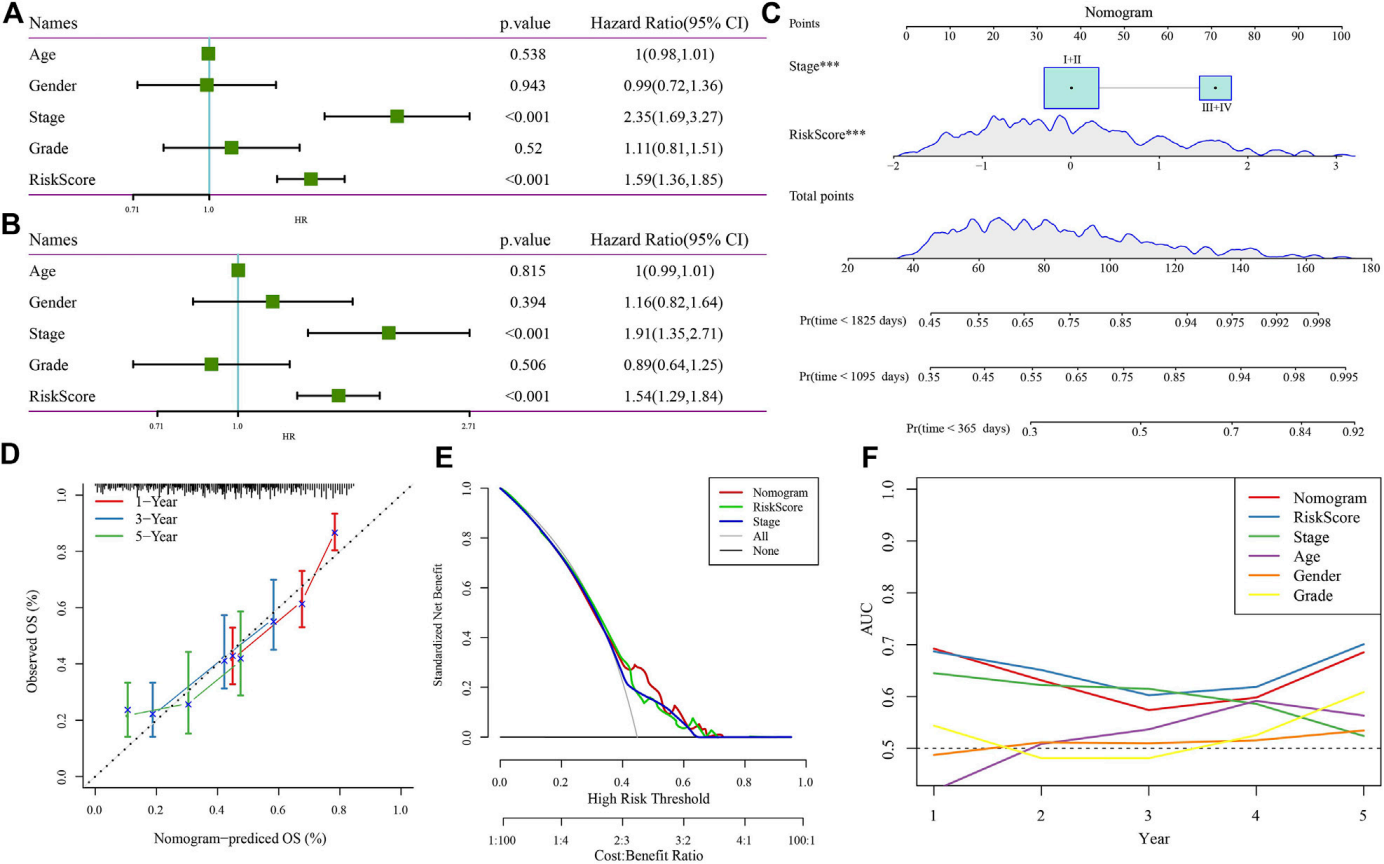
Nomogram construction and evaluation. Univariate **(A)** and multivariate **(B)** Cox regression analysis of the risk score, age, sex, stage, and grade. **(C)** Nomogram based on risk score and stage for predicting death rate at 1, 3, and 5 years. **(D)** Calibration curve for predicting the accuracy of the nomogram. **(E)** DCA plot of the nomogram, risk score, and stage. **(F)** AUC of the nomogram, risk score, and clinical features.

After demonstrating the robustness and reliability of the four-gene risk model related to RNF157-AS1, we next tried to further increase the accuracy of its application in the clinical setting. Cox regression analysis of the clinical features and risk score showed that only stage and risk score were independent risk factors of HCC prognosis (Figures 5A,B). Therefore, we used these factors to establish a nomogram for predicting 1-, 3-, and 5-year death rates (Figure 5C). The calibration curve showing the predicted overall survival was similar to the observed values (Figure 5D). Decision curve analysis (DCA) further demonstrated that patients could benefit more from the nomogram compared to other indicators (Figure 5E). Finally, ROC analysis revealed the superior performance of the risk score and nomogram for predicting long-term overall survival (Figure 5F).

## Discussion

Trans-arterial chemoembolization (TACE), an effective strategy for patients with unresectable HCC, requires the injection of embolizing agents in combination with doxorubicin (Fong and Tanabe, 2014). TACE is a recognized management scheme for patients with intermediate-stage HCC (Bruix and Sherman, 2011). The combination of the embolic effect with doxorubicin suppressed tumor progression and improved the prognosis of patients with HCC in randomized clinical trials (Llovet et al., 2002; Malagari et al., 2010). Moreover, doxorubicin-based TACE has a positive effect on reducing tumor size, which provides conditions benefitting liver transplantation (Fong and Tanabe, 2014). Nevertheless, resistance to doxorubicin markedly limits the treatment efficiency of patients with HCC, with only 27% of tumors treated with TACE showing a complete response and approximately 50% showing no response (Lammer et al., 2010). Consequently, it is of great importance to understand the mechanisms of resistance to doxorubicin in HCC. As a novel gene regulator, lncRNA is closely related to the occurrence, development, and prognosis of human disease, especially cancer. The abnormal expression of some lncRNAs may be related to the overgrowth, apoptosis inhibition, invasion, metastasis, and poor prognosis of HCC cells (Li et al., 2018; Pan et al., 2019; Huang et al., 2020). The present study is the first to comprehensively analyze the potential lncRNAs and genes associated with doxorubicin resistance in HCC.

In this study, we compared RNA-seq data from HCC samples and doxorubicin-resistant HCC samples to control data to identify key lncRNAs. Differential analysis between HCC and paracancerous samples and between doxorubicin-resistant and control samples revealed three candidate DElncRNAs. However, only RNF157-AS1 was an independent risk factor significantly associated with overall survival in HCC. Functional analysis revealed that some metabolic pathways, including bile acid metabolism, fatty acid metabolism, and oxidative phosphorylation, were negatively correlated with RNF157-AS1 expression. These metabolic pathways are involved in the pathogenesis or immune response of HCC (Wang et al., 2016; Ma et al., 2018; Liu et al., 2020); thus, RNF157-AS1 may regulate HCC progression and induce doxorubicin resistance by interacting with these metabolic pathways.

RNF157-AS1 has not been specifically reported in HCC or other cancer types. Some studies focusing on constructing gene signatures for cancer have identified RNF157-AS1 as a prognostic lncRNA in the signature. For instance, Lin et al. (2021) identified a five-lncRNA signature including RNF157-AS1 for predicting ovarian cancer prognosis. Jiang et al. (2020) reported RNF157-AS1 as a candidate gene related to HBV-based HCC.

To understand the potential mechanism of RNF157-AS1 in HCC development, we identified a series of RNF157-AS1-associated PCGs. Function analysis showed significant enrichment of the mRNA surveillance pathway, RNA transport, and the cell cycle in RNF157-AS1-associated PCGs, implying that RNF157-AS1 may be involved in regulating mRNA-related biological processes and cell proliferation. Based on these RNF157-AS1-associated PCGs, we constructed a risk model containing four prognostic PCGs (*CENPP*, *TSGA10*, *MRPL53*, and *BFSP1*). The four-gene risk model showed reliable performance and a high AUC score in different datasets. The result also showed that the risk score was an independent indicator of overall survival in HCC. In addition, we established a nomogram based on the HCC risk score and stage to increase the accuracy in the prediction of HCC prognosis. Compared to other indicators, the nomogram showed superior performance in the DCA plot, indicating that the nomogram could benefit patients with HCC.

Among these four genes, only *TSGA10* has been reported relatively often in cancer; the other three genes were less often reported. Tanaka et al. (2004) observed TSGA10 overexpression in 4 of 20 patients with HCC, two of whom showed antibodies against recombinant TSGA10 protein. TSGA10 was also suggested as a potential biomarker in cancer tumorigenesis (Mobasheri et al., 2007). Dianatpour et al. (2012) observed upregulated TSGA10 expression in breast cancer cell lines and suggested its important role in breast cancer proliferation and prognosis. However, the association between RNF157-AS1 and these four genes requires further analysis. The four-gene risk model also requires further verification in clinical cases. However, this study has several limitations. For example, the sample size of the included dataset is limited, which may lead to bias in the screening results and biological function analysis. Moreover, the prognostic model requires further experimental verification.

## Conclusion

In conclusion, the results of this study identified a key lncRNA (RNF157-AS1) that may contribute to doxorubicin resistance by involving metabolic pathways in HCC. Based on RNF157-AS1-associated PCGs, we constructed a four-gene risk model that reliably predicted HCC prognosis.

## Data Availability

The datasets presented in this study can be found in online repositories. The names of the repository/repositories and accession number(s) can be found in the article/Supplementary Material.
