# Supplementary figures and images for: Exploring a four-gene risk model based on doxorubicin resistance-associated lncRNAs in hepatocellular carcinoma
Source: Front Pharmacol. 2022 Nov 10;13:1015842. doi: 10.3389/fphar.2022.1015842 (PMC9708384; doi:10.3389/fphar.2022.1015842)

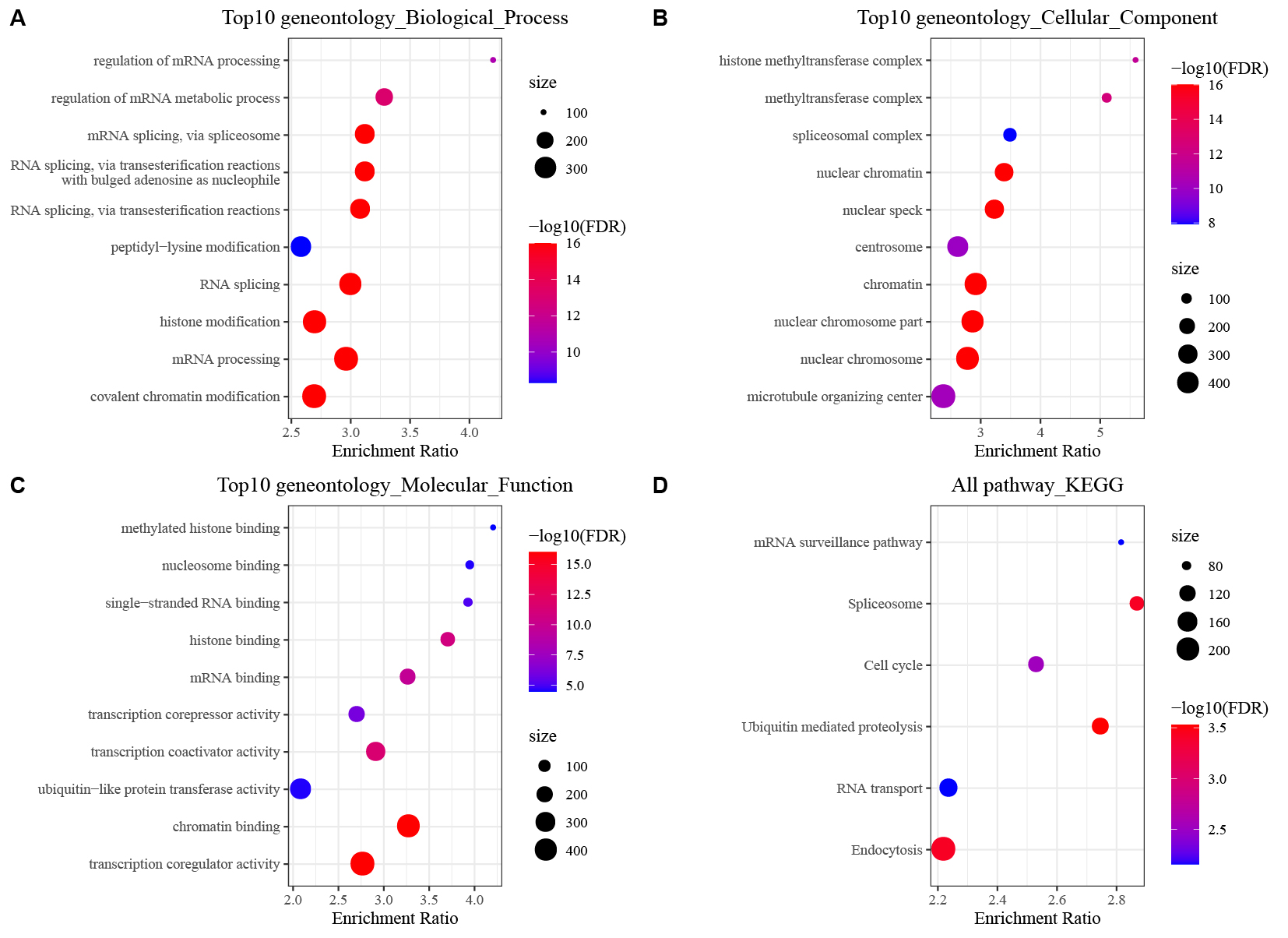

Supplement: Supplementary file 1 [file Image3.JPEG]

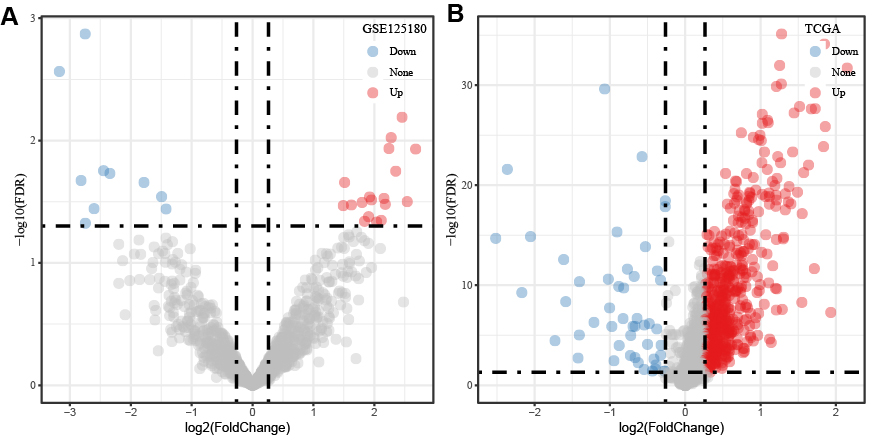

Supplement: Supplementary file 3 [file Image1.JPEG]

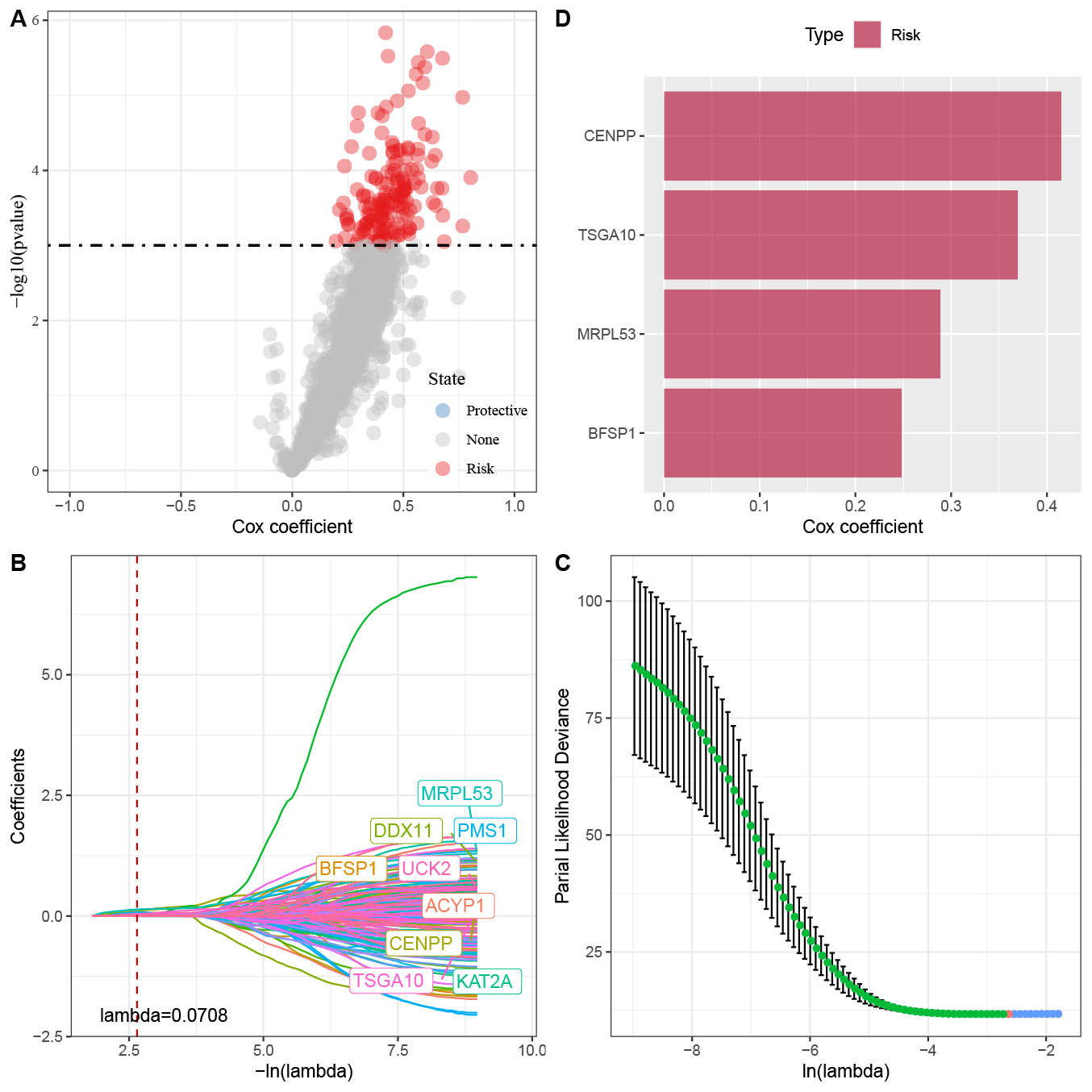

Supplement: Supplementary file 4 [file Image4.JPEG]

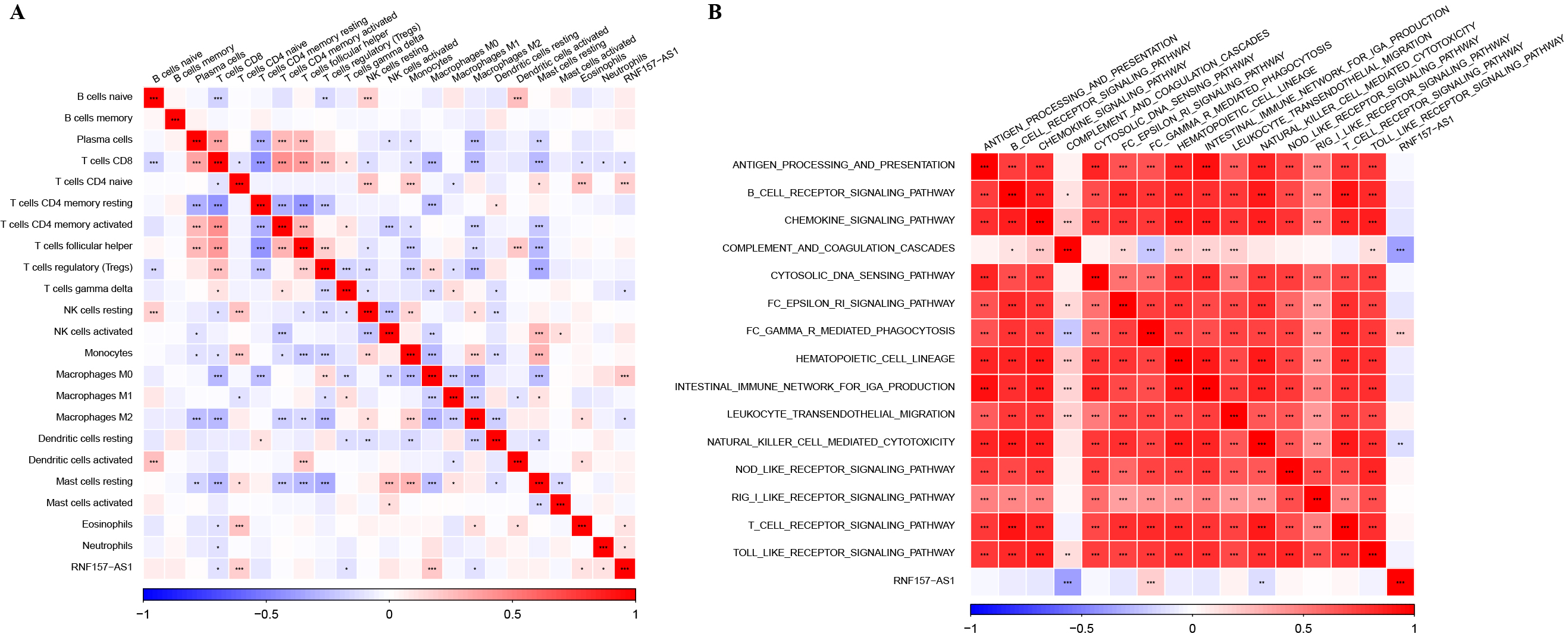

Supplement: Supplementary file 5 [file Image2.JPEG]
