# Supplementary material for: Exploring a four-gene risk model based on doxorubicin resistance-associated lncRNAs in hepatocellular carcinoma
Source: Front Pharmacol. 2022 Nov 10;13:1015842. doi: 10.3389/fphar.2022.1015842 (PMC9708384; doi:10.3389/fphar.2022.1015842)
Supplement: Supplementary file 2 [file Table1.DOCX]

Supplementary materials

Figure S1. Volcano plots of DElncRNAs in TCGA and GSE125180 datasets.

Figure S2. (A) The relation between RNF157-AS1 expression and immune infiltration. (B) The relation between RNF157-AS1 expression and immune-related pathways. Red and blue indicate positive and negative correlations respectively.

Figure S3. Functional analysis of RNF157-AS1-related genes. (A-C) The top 10 significantly enriched GO terms of RNF157-AS1-related genes. (D) The significantly enriched KEGG pathways of RNF157-AS1-related genes.

Figure S4. Construction of a risk model. (A) Volcano plot of 166 prognostic genes related to RNF157-AS1. (B-C) LASSO Cox regression analysis on 166 genes. Dashed red line indicates lambda value = 0.0708. (D) The coefficients of four prognostic genes.
