# Supplementary material for: Exploring a four-gene risk model based on doxorubicin resistance-associated lncRNAs in hepatocellular carcinoma
Source: Front Pharmacol. 2022 Nov 10;13:1015842. doi: 10.3389/fphar.2022.1015842 (PMC9708384; doi:10.3389/fphar.2022.1015842)
Supplement: Supplementary file 6 [file Table2.DOCX]

For the data analyzed in this study please see:

https://www.jianguoyun.com/p/DV-sy50QharcChjk8s8EIAA
